# 

**Published:** 1846-10-01

**Authors:** 


Quain on the Arteries.

Our readers will observe, among the Advertisements, the announcement of a great reduction in the price of this valuable work. It is an honour to the age and country, and we are much pleased to find it thus brought within the means of private individuals, as well as public institutions.

### BIBLIOGRAPHICAL RECORD.

1. The Economy of the Animal Kingdom considered Anatomically, Physically, and Philosophically. By *Emanuel Swedenborg.* Translated from the Latin, by the Rev. *Augustus Clissold,* M.A. Two vols. 8vo. London, 1846.

2. The Surgical, Mechanical, and Medical Treatment of the Teeth, including Dental Mechanics. By *James Robinson.* Illustrated with one hundred and thirty-nine engravings. Small 8vo, pp. 340. London, 1846.

3. Dr. Hooper's Physician's Vade-mecum, or a Manual of the Principles and Practice of Physic. New edition, considerably enlarged and improved, with an Outline of General Pathology and Therapeutics. By *W. A. Guy,* M.B. Foolscap 8vo, pp. 531. London, 1846.

*This edition is enlarged and improved. It forms an exceedingly good manual of theoretical and practical medicine for practitioners as well as students.*

4. On the Sanative Influence of Climate. By Sir *James Clark,* Bart, M.D. F.R.S., &c. Fourth edition. 8vo, pp. 431. London, 1846.

5. The Retrospect of Medicine; being a half-yearly Journal, containing a Retrospective View of every Discovery and Practical Improvement in the Medical Sciences. By *W. Braithwaite.* Vol. XIII. Pp. 530. London, 1846.

6. A Guide to the Use of the Buxton Waters. By *William H. Robertson,* M.D. Third Edition, foolscap 8vo, pp. 32. London, 1846.

*Useful to the invalid and the physician.*

7. Practical Observations on Mineral Waters and Baths, with Notices of some Continental Climates, and a Re-print of the Cold Water Cure. By *Edwin Lee,* Esq., Small 8vo, pp. 182. London, 1846.

*In our next.*

8. Mesmerism in India, and its practical Application in Surgery and Medicine. By *James Esdaile,* M.D., Civil Assistant-Surgeon H.C.S. Bengal, Foolscap 8vo, pp. 318. London, 1846.

9. The American Journal of Insanity. Edited by the Officers of the New York State Lunatic Asylum. Utica, April 1846. 8vo.

10. Third Annual Report of the Managers of the State Lunatic Asylum, made to the Legislature Jan. 23d, 1846. Albany.

11. The Medical Examiner and Record of Medical Science. Edited by *Robert M. Huston*, M.D. New Series, January to May, 1846. Philadelphia.

12. Quelques Considerations en Reponse a l'Examen de la Phrenologie de M. le Professeur Flourens. Par M. *S. de Wolkoff.* 1846.

13. Outlines of Structural and Physiological Botany. By *Arthur Henfrey*, F.L.S., &c. Part I. 1846. *In our next.*

14. The Brain and its Physiology ; a Critical Disquisition on the Methods of determining the Relations subsisting between the Structure and Functions of the Encephalon. By *Daniel Noble*, Member of the Royal College of Surgeons. Small 8vo, pp. 466. London, 1846.

15. Transactions of the Medical and Physical Society of Bombay. No. VII. Octavo, pp. 206. Bombay, 1844.
*In our next.*

16. On the Antidotal Treatment of the Epidemic Cholera. By *John Parkin*, M.D., &c. Octavo, pp. 60. London, 1846.

17. The Microscopic Anatomy of the Human Body in Health and Disease; illustrated with numerous Drawings in Colour. By *Arthur Hill Hassall*, Author of the British Fresh-water Algæ, &c. Parts 1 and 2. Octavo. Highley, London, 1846.

18. The Use of the Body in Relation to the Mind. By *George Moore*, M.D. Octavo, pp. 437. London, 1846.
*In our next.*

19. Elements of Physics. By *C. F. Peschel.* Translated from the German, with Notes, by *E. West*. Illustrated with Diagrams and Woodcuts. Parts II. and III. Imponderable Bodies. 12mo. London, 1846.

20. A New Universal, Etymological, and Pronouncing Dictionary of the English Language, embracing all the Terms used in Art, Science, and Literature. Parts I. to IX. Royal 8vo. London, Gilbert, 1846.
*A work of immense labour, and one which promises to be of very great utility.*

21. On Wounds and Injuries of the Arteries of the Human Body, with the Treatment and Operations required for their Cure. Illustrated by 130 Cases, selected from the Records of the Practice of the most celebrated Surgeons in Europe and America, with the Critical Remarks of the Author on each. By *G. J. Guthrie*, F.R.S. Royal 8vo, pp. 97. London, 1846.

22. Fever physiologically considered—Considerations on Yellow Fever, Typhus Fever, Plague, Cholera, and Sea Scurvy; also the Questions of Contagion and the Quarantine Laws, with an Address to the Public, and on the Popular Treatment of Cholera. By *David M'Connell Reed*, Esq. 12mo, pp. xli.—262. London, 1846.

23. The Why and the Wherefore, or the Philosophy of Life, Health, and Disease ; New and Original Views explanatory of their Nature, Causes, Connexion, &c.—the Fruit of 30 years' observation and experience. By *Charles Searle*, M.D., &c. Octavo, pp. 296. 1846.

24. Reports of the Officers of the Retreat for the Insane at Hartford, Conn. 1843-4-6. 8vo, pp. 108.

25. The Nerves of the Uterus. By *Thomas Snow Beck*, Esq. Surgeon. From the Philosophical Transactions, Part II., for 1846. 4to, plates.

26. Cosmos : Sketch of a Physical Description of the Universe. By *Alexander Von Humboldt.* Vol. I. Translated under the Superintendence of Lieut.-Col. *Edward Sabine*, R.A., &c. 8vo, pp. 497. London, 1846.
*In our next.*

27. A System of Surgery. By *J. M. Chelius*, Translated from the German, and accompanied with additional Notes and Observations. By *John F. South*, Professor of Surgery to the Royal College of Surgeons of England. 8vo, parts to 13. London, 1846.

28. Clinical Collections and Observations in Surgery, made during an attendance on the Surgical Practice of St. Bartholomew's Hospital. By *W. P. Ormerod*, Fellow of the Royal College of Surgeons of England, late House-Surgeon at St. Bartholomew's Hospital. 8vo, pp. 324. London, 1846.
*In our next.*

29. An Easy Introduction to Chemistry. By *George Sparkes*, late Madras Civil Service. Second Edition, 12mo, pp. 190. London, 1846.
*A most useful guide for those who may wish to perform the various elementary chemical experiments at a trifling cost.*

30. Medical Report of the House of Recovery and Fever Hospital, Cork Street, Dublin, for two years, from 1st January, 1844, to 31st Dec. 1845. By *George Kennedy*, M D. 8vo, pp. 68. Dublin.

31. A Medical Topography of Tunbridge Wells. By *R. H. Powel*, M.B., M.D. Tunbridge, 1846. 12mo, pp. 174.

32. Annual Report of the Cork Fever Hospital and House of Recovery for the Year 1845. 8vo, pp. 16. Cork.

33. The Liverpool Health of Towns Advocate, Nos. 1 to 13. Edited by *John Sutherland*, M.D. 8vo. pp. 124.

34. Notes on the Epidemic Cholera. By *R. H. Kennedy*, M.D., &c. Second Edition, small 8vo, pp. 279. London, 1846.
*In our next.*

35. Experimental Researches on the Food of Animals and the Fattening of Cattle, with Remarks on the Food of Man. Small 8vo, pp. 195. London, 1846.
*In our next.*

36. Revue Medicale, Feb. March, April, 1846.

37. Dublin Medical Review, Nos. 1, 2, and 3.
*In exchange.*

38. Edinburgh Medical Journal, July.
*In exchange.*

39. British and Foreign Medical Review. July.
*In exchange.*

40. Medical Gazette, July to October.
*In exchange.*

41. Gazette Medicale, January to October.
*In exchange.*

42. The American Journal of the Medical Sciences. Edited by *Isaac Hays*, M.D. July, 1846. *In exchange.*

### INDEX.

Abscess from pblebitis ............ 165
Abscess, treatment of milk ........ 185
Abscess, pelvic .................. 260
Abscess, opening by incision........ 426
Abscess, hepatic .................. 370
Abscess, hepatic, treated by mercury 375
Abscess after fracture or dislocation.. 541
Aged, cause of death of the ........ 540
Amaurosis, traumatic............... 544
Amenorrhœa, arterial murmurs in .. 346
Ammonia, phosphate of............ 264
Amputation, question of immediate.. 93
Amputation, question of, in gun-shot wounds ....................... 95
Anæmia, arterial murmurs in ...... 339
Analysis, Will on qualitative........ 237
Analysis, objects of.............. ... 488
Anatomy, pathological, Hasse & Grosse 161
Anatomy, Hassall on microscopic.... 523
Aneurism, murmur in ............. 337
Ani, sphincter, division of.......... 265
Anus, fissure of the ............... 266
Aorta, insufficiency of valves of .... 339
Aphides, generation of ............ 23
Apoplectic constitution, the........ 42
Apoplexy, nature of .............. 41
Apoplexy, connection of, with disease of the heart..................... 42
Apoplexy, period of life prone to.... 44
Apoplexy, treatment ............... 44
Apoplexy, pulmonary.......... ... 175
Arterial murmurs, Beau on ........ 333
Arteries, inflammation of .......... 167
Arteries, murmur of ............... 333
Arteries, Porta and Guthrie on...... 464
Arteries, anatomy of the .......... 465
Arteries, effects of ligature on ...... 467
Arteries, torsion of................ 469
Arteries, consequence of obliteration 470
Arteries, wounds of ............. 475
Ashley, Lord, on the insane ........ 55
Assimilation, Mialhe on............ 281
Asylums, management of .......... 394
Asylums, site and construction of.... 397
Asylums, ventilation of............ 399
Asylums, attendants of ............ 399
Asylums, quiet of the European ... 400
Auscultation, Laennec on .......... 238
Beau on arterial murmurs.......... 333
Beck on uterine nerves ............ 514
Becquerel and Rodier on the blood .. 533
Bee, nurse-bee, economy of ........ 127
Bile, colouring matters of....... .. 485
Bile, Liebig's views of, disputed...... 487
Bile, chemistry of the ............ 495
Blood, changes in inflammation...... 483
Blood, coloring matter of .......... 484
Blood, corpuscular bodies of ........ 489
Blood, chemical relations of........ 491
Blood, analysis of healthy .......... 493
Blood, the condition of morbid...... 494
Boil, treatment of ................ 390
Bone on yellow fever.............. 210
Bouillaud, Nosographie Medicale .... 187
Bowditch' Young Stethoscopist...... 240
Brain, circulation of the .......... 35
Brain, influence of vascular pressure on 37
Brain, disease of, from disease of heart 46
Brain, Noble on physiology of the .. 447
Brain, Gall's anatomy of the........ 449
Brain, modes of study of its physiology 454
Breast, cero-cystic tumours of the .. 10
Breast, question of operating for cancer of the ..................12, 268
Breast, Tuson on the female ........ 183
Breast, traumatic suppression of milk 185
Breast, neuralgia of the............ 269
Breast, application in cancer of..186, 556
Briggs on stricture................ 230
Brodie, Sir B. lectures of .......... 1
Bronchi, dilatation of the .......... 179
Broussais, sketch of .............. 171
Burrows on disorders of the cerebral circulation ........ ........... 34
Cæsarian operation, theology of .... 557
Cachexiæ, arterial murmurs in...... 343
Cachexiæ, agency of iron in........ 345
Calmeil, De la Folie .............. 49
Calomel, importance of, as a remedy.. 513
Cancer of the lungs .............. 183
Cancer of the breast ..............
Cancer of the breast, relapses of .... 552
Carbon, chloride of............. .. 186
Carotids, effects of ligature of the .. 39
Carotids, ligature of both.......... 376
Carpenter, manual of physiology... 122
Carus, cranioscopy of.............. 453
Catarrh, senile . ................. 177
Caustics, Brodie on .............. 18
Cerebellum, functions of the ...... 456
Chemistry, animal..... .......... 481
Chervin, sketch of ................ 174
Children, syphilis in .............. 14
Children, mortality of male ........ 280
Children, Coley on diseases of ...... 386
China, medical notes on............ 73
China, state of medicine in ........ 90
Chlorosis, arterial murmurs in...... 342
Chlorosis, Gintrac on ............ 556
Cholera, epidemic, Parkin on........ 559

Chorea, electrical .... ........... 545
Chusan, diseases of.............74, 79
Circulation, the pulmonic.......... 71
Circulation, the collateral......... 470
Clark on Climate ............... 520
Clavicle, fracture of ............. 548
Clutterbuck on inflammation........ 235
Coley on diseases of children ...... 386
Coli-caput, inflammation of the .... 369
Colostrum, Simon on the .......... 496
Contagion, Fergusson on .......... 211
Convalescence, management of...... 156
Costello on lunatic asylums ........ 58
Cranioscopy of Carus.............. 453
Croup, surgical treatment of........ 262
Day on animal chemistry .......... 481
Demoniacal possessions ........51, 417
Dentition, Coley on .............. 388
Dentists, false position of .......... 405
Dentists, American................ 406
Deontology, medical, Simon on .... 431
Diatheses, the seven .............. 270
Diseases, Bouillaud's classification of 193
Dissecting-rooms, purification of .... 272
Dropsies, arterial murmurs in ...... 348
Drowning, death from ........... 7
Dupuytren, sketch of.............. 168
Dysentery in China.............75, 83
Dysentery in connection with hepatic abscess......................... 372
Dysentery, pathology of............ 376
Dysentery, scorbutic .............. 379
Dysentery, treatment of............ 379
Dysentery in children.............. 391
Eclair fever, history of the.......... 219
Eclectism in medicine ............ 152
Education, medical, Brodie on ...... 2
Education, medical, necessity of its improvement .................... 510
Egypt, sanative climate of.......... 522
Elephantiasis in China ............ 80
Emphysema of the lungs .......... 180
Endocarditis, Hope on ............ 169
Entozoa, reproduction of .......... 29
Epidemics, characteristics of........ 300
Epilepsy, opium in................ 368
Epistaxis, compression of carotids in 282
Epistaxis, plugging the nares in .... 392
Erysipelas of children............. 389
Erysipelas, nitrate silver in ........ 548
Esdaile on mesmerism ............ 558
Factories, influence on scrofula...... 141
Fergusson, recollections of a professional life......................... 210
Fever, remittent, of China.......... 82
Fever, remittent, of India .......... 358
Fever, intermittent, of China and India.......................83, 358
Fever, intermittent, Bouillaud on .. 215
Fever, simple, Bouillaud on ........ 197
Fever, continued, in India.......... 366
Fever, typhoid...............201, 273
Fever, typhus.................... 204
Fever, typhus, contagion denied .... 211
Fever, yellow ...............206, 210
Fever, yellow, non-contagion of .... 214
Fever, the Eclair ................. 218
Fever, contagion of puerperal ...... 267
Fever in Ireland.................. 559
Fevers, considered as phlegmasiæ.... 195
Fevers, arterial murmurs in........ 347
Fibula, fracture of ................ 258
Flogging, military cruelties of .. ... 110
Flourens on phrenology .......... 447
Foot, bleeding from .............. 423
Forbes on homœopathy............ 501
Fracture, treatment of ununited .... 8
Fracture, compound .............. 92
Fracture, law of formation of abscess after ......................... 541
Furunculus, treatment of .......... 390
Gall, value of the cerebral anatomy of 449
Gall and Spurzheim, fallacies of .... 456
Gangrene, senile..............20, 168
Gangrene after ligature of arteries .. 474
Gastric juice, nature of............ 495
Gastrodynia, treatment of.......... 418
Geddes, diseases of India .......... 355
Generation, alterations of .......... 22
Glottis, inflammation and œdema of.. 177
Gonorrhœa, Ricord on ............ 553
Gout, phosphate of ammonia in .... 264
Griffith on animal chemistry........ 481
Gross, pathological anatomy........ 161
Guthrie, wounds of arteries ........ 495
Hæmoptysis, Hope on ............ 176
Hæmorrhoids, nature of .......... 166
Hæmostatics, Velpeau on .......... 552
Hair, analysis of.................. 498
Hanging, death from.............. 4
Harveian Oration .. ............. 557
Hassall, microscopical anatomy .... 523
Hasse, pathological anatomy........ 161
Health, equilibrium of ............ 149
Heart, diseases of, as causes of cerebral affections.................. 43
Heart, pathological changes in...... 169
Heart, size of vessels of . ........ 176
Heart, disease of, in relation to hepatic disease......................... 172
Heart, pneumonia, supervening in .. 172
Heart, the fatty .................. 173
Heart, inflammation of, as a cause of fever . ....................... 197
Heat, influence on vitality.......... 127
Hemiplegia, treatment of .......... 44
Hernia humoralis, treatment of .... 100
Hernia, inguinal.................. 429
Hernia, femoral .................. 430
Hernia, taxis in .................. 429
Hindu system of medicine.......... 411
Hoffman on physiological chemistry.. 481

Homœopathy, Forbes on .......... 501
Hong-Kong, insalubrity of ......... 81
Hypochondriasis, arterial murmurs in 349
Imagination, cause of scientific progress 156
India, Geddes & Parkes on diseases of, 355
Inflammation, Clutterbuck on ...... 235
Insane, condition of the............ 53
Insane, management of.......... 64, 394
Insane, large dormitories for........ 398
Insane, exercise & amusements of, 64, 401
Insane, attendants of ............... 399
Insane, diet of the ................. 65
Insane, religious instruction for.. 66, 403
Insane, schools for the.......... 67, 402
Insane, hospitals for the............ 392
Insane, paralysis of the............ 549
Insane, state management of........ 56
Insanity, statistics of.............. 58
Insanity, recoveries and mortality .. 59
Insanity, liability to, as to sex ...... 60
Insanity, liability of various ages to.. 62
Insanity, duration of .............. 63
Insanity, treatment of........... 65, 403
Insanity, use of restraint in...... 66, 401
Insanity, influence of phrenology on 461
Iodine, endermic use of............ 282
Iris, anæsthesia of ................ 259
Kramer, contributions to aural medicine ........................ 240
Laennec, Herbert's translation of.... 239
Larrey, sketch of .... ............. 176
Lead, plates of, for wounds ........ 160
Lee, Mr. on medical organization .. 209
Lee, Dr. on the uterine nerves ...... 514
Leeson on Liebig.................. 501
Leprosy among the Hindoos ........ 417
Ligature, Porta on the .......... .. 467
Light, influence on animal life ...... 126
Lisfranc on amputation............ 95
Liston on practical surgery ........ 419
Lithotomy, Liston and Malgaigne on, 425
Lithotrity, Liston on.............. 425
Liver, disease of, in relation to heart-disease........................ 172
Liver, inflammation and abscess of .. 37
Liver, abscess of, in connection with dysentery .................... 372
Lunacy act, the .................. 54
Lunatics, state, management of .... 56
Lungs, the circulation through...... 71
Lungs, cancer of the.............. 182
Lungs, emphysema of the.......... 180
Lymphatics, diseases of............ 163
Malaria in ships' holds . ........ 77, 91
Malgaigne, operative surgery ...... 419
Manure collected in China ........ 79
Marshall, military miscellany ...... 107
Matter, affinity of organic & inorganic 123
Medical profession, moral duties of 433, 445
Medical studies, material nature of .. 437
Medicine, eclectism in ............ 153
Medicine, Bouillaud on the progress of 189
Medicine, Hindu system of ........ 411
Medusæ, generation of the ........ 25
Melancholic temperament.......... 165
Mendelssohn on respiration ........ 68
Meningitis, cerebro-spinal.......... 103
Menstruation, arterial murmurs in .. 346
Mesmerism in India .............. 558
Mialhe on assimilation ............ 281
Midwifery statistics .............. 260
Mortification, Brodie on ......... 18
Mortification, spontaneous dry... .. 76
Muscle, composition of............ 499
Myopia, case of .................. 259
Neck, wounds of, suture in ........ 101
Nerves, influence on organic functions 69
Nerves, affections of 3rd and 5th pairs, 542
Nerves, sympathetic, relations of.... 517
Nerves, the uterine, size of ........ 518
Neuralgia, facial.................. 542
Noble on physiology of the brain.... 447
Numerical system, fallacies of ...... 105
Ophthalmia, turpentine in ........ 257
Orchitis, treatment of ............ 100
Organization, theory of............ 125
Paralysis, local, from pressure ...... 282
Paralysis, case of.................. 455
Paralysis of the third pair.......... 543
Paralysis of the insane ............ 549
Paris, public health of ............ 278
Parise on health and disease........ 148
Parkes on Indian dysentery ........ 355
Patella, fracture of the ............ 540
Pelvis, abscess of the .............. 260
Pericarditis in brain affections ...... 47
Pericarditis, Hasse on ............ 169
Periodicals, French & English Medical, 274
Pertussis, treatment of ............ 391
Pharynx, granular disease of........ 276
Phillipps on scrofula .............. 128
Phillips on the Pharmacopœias, &c... 360
Phlebitis, Hasse on................ 164
Photophobia, treatment of.......... 538
Phrenology, fallacies of ............ 457
Phrenology relative to insanity...... 461
Phthisis among soldiers............ 105
Phthisis, question of identity with scrofula ...................... 132
Phthisis, Rainey on pathology of.... 182
Phthisis, change of climate in ...... 520
Physicians, College of, inertness of .. 436
Physiology, mode of investigating cerebral ....................... 451
Physiology illustrated by pathology .. 455
Plague, the Report of the French Academy on the ................... 286
Plague, countries in which it is indigenous ....................... 290
Plague, predisposing & exciting causes 293
Plague, endemic in Egypt .......... 296
Plague, epidemic character of. ...... 298

Plague, difference between the epidemic and sporadic .................... 305
Plague, mode of diffusion .......... 306
Plague, inoculation of the.......... 308
Plague, mode of transmission ....... 310
Plague, non-transmissibility of, by merchandize ..................... 323
Plague, not epidemic in Europe...... 327
Plague, incubation of the...... 329, 527
Pleurisy, pathological changes in .... 173
Pleurisy, paracentesis in acute ...... 279
Pneumonia of drunkards .......... 175
Pneumonia, catarrhal.............. 178
Polyæmia, serous ................. 340
Porrigo, treatment of.............. 390
Porta on the arteries .............. 464
Pregnancy, arterial murmurs in ..... 347
Pregnancy, longings in ............ 414
Protein, medical uses of............ 184
Protein, oxides of ................ 482
Prus' Report on the Plague ........ 286
Puerperal fever, contagion of ....... 267
Puerperal state, management of the.. 278
Purpura, arterial murmurs ......... 348
Pus, arthritic .................... 496
Pus, tests of ..................... 497
Quackery, to be discountenanced by the profession.................. 506
Quain on the arteries.............. 561
Quarantine, Report of the French Academy on ........................ 286
Quarantine, changes proposed in .... 331
Quarantine, correspondence relating to 525
Radius, fracture of.... .......... 257
Ray on hospitals for the insane ..... 392
Reed on fever..................... 559
Remedies, Brodie on new .......... 16
Rheumatism, phosphate of ammonia in 264
Rheumatism, quinine in........ 267, 282
Rheumatism, carditis in............ 282
Rheumatism in India.............. 382
Ringworm, treatment of ........... 390
Robinson on the teeth ............. 404
Salpæ, generation of ......... .... 27
Scorbutus, arterial murmurs in .... 348
Scrofula, nature of............... 129
Scrofula, microscopic and chemical characters .................... 131
Scrofula, condition of the blood in .. 131
Scrofula, identity with phthisis. .... 132
Scrofula, rarity in England......... 137
Scrofula, etiology of .............. 137
Scrofula, influence of occupation .... 141
Scrofula, influence of climate ....... 145
Scrofula, treatment of ............. 147
Searle on life, health, and disease .... 501
Seasons, medical constitution of the.. 208
Silver, nitrate, local use of.......... 548
Simon on animal chemistry ........ 481
Simon, Dr. Max., on medical deontology 431
Small-pox, history of.............. 416
Soldiers, phthisis among .......... 105
Soldiers, diet of, in India .......... 356
Spleen, diseases of, in India ....360, 369
Sprains, treatment of.............. 97
Steenstrup on alternations of generations.......................... 22
Strabismus, case of accidental ...... 259
Strangulation, death from.......... 4
Stricture, Briggs on .............. 230
Surgery, operative, defective instruction in ........................ 420
Surgery, Liston and Malgaigne on .. 419
Swedenborg's Economy of the Animal Kingdom ...................... 478
Sympathetic nerve, relations of...... 517
Synochus on angeio-carditis ........ 197
Syphilis, Brodie on use of mercury in, 13
Syphilis in children .............. 14
Teeth, Robinson on ............... 404
Teeth, irregularities of ........... 409
Teeth, caries of .................. 410
Thorax, vibrations of.............. 282
Thurnam on statistics of insanity.... 58
Tibia, chronic abscess ............. 21
Tic-douloureux, Brodie on.......... 15
Torsion, Porta on ................ 469
Tubercle, Rainey on .............. 182
Tumours, sero-cystic, of the breast .. 10
Tumours, fatty, Brodie on.......... 16
Tuson on female breast ............ 183
Ulcers, sloughing, in China .......75, 87
Ulcers dressed by leaden plates...... 160
Ulcers, rheumatic ................. 383
Urethra, dimensions of ............ 231
Urine, retention of............ 235, 555
Urine, colouring matters of ........ 485
Urine, pathological relations of...... 497
Urine, diabetic .................. 497
Uterus, Lee & Beck on nerves of .... 514
Uterus, flexions and engorgements of, 548
Vagina, dilatability of............. 431
Varix, aneurismal murmurs of...... 337
Vein, jugular, murmurs of...... .... 338
Veins, Hasse on inflammation of .... 164
Veins, dilatation of................ 166
Veins, varicose, treatment of ....... 11
Venesection from the foot .......... 423
Vesication, extemporaneous ........ 282
Vitality, nature of the phenomena of.. 123
Vitality, connection with organization 124
Vitality, influence of light and heat on 126
Vivisections, unnecessary, condemned, 443
Walker on compound fracture ...... 92
Will on qualitative analysis ........ 237
Wilson's Medical Notes on China.... 73
Winslow on the Lunatic Act........ 53
Witchcraft, the insane accused of.... 50
Wolkoff on phrenology ............ 447
Worms, intestinal, in China......... 87
Wounds healed by lead plates ....... 160
Wounds healed by cupping-glasses ... 163

